# APMAP Promotes Epithelial-Mesenchymal Transition and Metastasis of Cervical Cancer Cells by Activating the Wnt/β-catenin Pathway

**DOI:** 10.7150/jca.59595

**Published:** 2021-08-28

**Authors:** Xiuting Zhu, Zijin Xiang, Lingxiao Zou, Xueru Chen, Xiangdong Peng, Dabao Xu

**Affiliations:** 1 Department of Gynaecology, The Third Xiangya Hospital, Central South University, Changsha, 410013, Hunan, China.; 2 Department of Pharmacy, The Third Xiangya Hospital, Central South University, Changsha, 410013, Hunan, China.

**Keywords:** APMAP, cervical cancer, β-catenin, EMT

## Abstract

Cervical cancer is a malignant tumor of the female reproductive system. At present, its occurrence, development and transfer mechanism are not entirely clear. APMAP (Adipocyte Plasma Membrane Associated Protein) is a glycosyl type II transmembrane protein that is mainly distributed in the plasma membrane and endoplasmic reticulum of adipocytes. APMAP has been reported to be involved in lipid transport and can induce epithelial-mesenchymal transition of prostate cancer and the liver metastasis of colorectal cancer. However, the role of APMAP in cervical cancer is still unknown. We analyzed the expression and prognosis of APMAP using data in both the GEO and the TCGA databases. We analyzed the function of APMAP using Transwell, wound healing assay and flow cytometry, and assessed the main mechanisms of APMAP by RT-PCR and Western blotting. We found that APMAP was highly expressed in cervical cancer tissues, and patients with high expression had poor prognosis. The functional *in vitro* experiments demonstrated that APMAP knockdown significantly inhibited the migration ability of cervical cancer cells, but had little effect on cell apoptosis. Mechanically, APMAP promotes cervical cancer cell migration and epithelial-mesenchymal transition by activating the Wnt/β-catenin pathway. Overall, APMAP is a potential prognostic marker as well as a therapeutic target of cervical cancer.

## Introduction

Cervical cancer, which has seriously threatened their health and life, is the fourth most malignant tumor of women worldwide. In 2018, cervical cancer accounted for 310,000 deaths, 6.6% of cancer incidence and 7.5% of mortality worldwide 1. At present, cervical cancer treatment mainly consists of surgery, radiotherapy, chemotherapy and immunotherapy 2. In recent years, with the continued development of medical technology and the application of cervical cancer vaccine, the incidence and mortality rates of cervical cancer have decreased in developed countries. However, the incidence is still on the rise and it tends to be diagnosed in younger patients 3. The occurrence and development of cervical cancer are related to many factors, such as chromosome aberration, DNA mutation, abnormal methylation, abnormal pathway regulation, and so forth 4-7. Consequently, it is crucial to explore the potential mechanism by which related genes affect cervical cancer cells.

The Wnt/β-catenin signaling pathway is key pathways regulating tumor invasion and metastasis 8, 9. Numerous studies have shown that the Wnt signaling pathway plays an important role in the occurrence and invasion of cervical cancer 7, 10. The Wnt signaling pathway is associated with different proteins required for cell proliferation and differentiation. For example, SOX14 activates the Wnt/β-catenin signaling pathway, triggering downstream signaling cascades that promote the proliferation and invasion of cervical cancer cells 11. Overexpression of PIN1 can inhibit cell sensitivity to cisplatin by regulating the Wnt/β-catenin pathway 12. Additionally, when the Wnt/β-catenin pathway is activated, the stem-like properties associated with epithelial-mesenchymal transition (EMT) can be maintained in cervical cancer cells 13, 14. Studies suggest the potential research value of the Wnt/β-catenin pathway in cervical cancer.

APMAP, otherwise known as C20orf3, is a 46 kDa glycosyl-type II transmembrane protein mainly distributed in the plasma membrane and endoplasmic reticulum of adipocytes 15, 16. APMAP has been reported to have hydrolase activity and is expressed in various human tissues as well as various cell lines, especially in adipose tissue with a high expression level 17. Studies have shown that APMAP plays important biological roles in fat differentiation, cancer development, viral infections, Alzheimer's disease, and so on 15, 18-20. Albrektsen et al found that APMAP regulated adipocyte differentiation, and the silencing of APMAP in 3T3-L1 adipocytes could significantly reduce the differentiation ability of adipocytes while down-regulating the expression of PPARγ, C/EBPA, AP2 as well as other adipocyte markers 15. APMAP was found to be the target gene of PPARγ and could be directly regulated by PPARγ^21^. However, there is little on APMAP in cancer research. Jiang et al. revealed that cholesterol increased the stability of EGFR by enhancing the interaction between APMAP and EGFR substrate 15 associated protein (EPS15R), thereby inducing the epithelial mesenchymal transition of prostate cancer 19. Another clinical study noted that APMAP expression was higher in the tissues of colorectal cancer patients with liver metastasis than in patients without metastasis. This indicated that APMAP may be used as a biomarker to determine the prognosis of liver metastasis from colorectal cancer 17. However, it is unknown whether APMAP plays a role in cervical cancer.

Using bioinformatics and proteomic sequencing analysis, we found that APMAP may play an important function in cervical cancer. We also found that the expression of APMAP in cervical cancer tissues was higher than in normal tissues, and the high expression of APMAP had a poor prognosis. Our study demonstrated that APMAP may promote cervical cancer cell migration and epithelial-mesenchymal transition by activating the Wnt/β-catenin pathway. These results could potentiate the use of APMAP as a novel strategy in predicting the progression of cervical cancer.

## Methods

### Patient samples

Three pairs of cancerous tissue and paracancerous tissue samples were collected for proteomic analysis from patients diagnosed with cervical cancer and undergoing hysterectomies in the Third Xiangya Hospital of Central South University from the period of January 2017 to December 2017. The collection of specimens and subsequent experimental use were approved by the Medical Ethics Committee of the Third Xiangya Hospital of Central South University (Ethics No. 2019-S467).

### iTRAQ proteomic analysis

Quantitative iTRAQ-based proteomic analysis was performed by Gene Denovo Biotechnology Co. Ltd (Guangzhou, China). Total proteins were extracted from tissue samples and the protein concentration was determined using a BCA kit (Bio-Rad Laboratories, Hercules, CA) according to the manufacturer's instructions. 0.2 M Tris(2-Carboxyethyl) Phosphine (TCEP) was added to 100 μg of each protein and incubated at 55 °C for 1 h. Then, 375 mM iodoacetamide (IAA) was added to the samples and incubated in darkness for 30 min, after which the proteins were digested by trypsin with sequentially modified trypsin (Promega, Madison, WI). The digested peptides for each sample were labeled with iTRAQ according to the manufacturer's instructions. Peptides from each iTRAQ labeled sample were fractionated by high-pH separation using the Ultimate 3000 system (Thermo Fisher Scientific) connected to a reverse-phase column (XBridge C18 column, 4.6 mm × 250 mm, 5 μm; Waters Corporation) and subjected to capillary liquid chromatography‐tandem mass spectrometry (LC‐MS/MS) using an Easy-nLC 1000 system (Thermo Fisher Scientific) connected to a Q Exactive mass spectrometer (Thermo Fisher Scientific) equipped with an online nanoelectrospray ion source. The mass spectra were extracted by Mascot Distiller software version 2.6 and the original mass spectrum data were searched by database using Mascot (Matrix Science, London, UK; version 2.5.1) 22.

### GEO database and TCGA database analysis

We downloaded the GSE9750 and GSE7803 datasets from the GEO database (http://www.ncbi.nlm.nih.gov/geo/) and the CESC dataset from the TCGA database (https://cancergenome.nih.gov/abouttcga/overview). GSE9750 included 33 primary tumors and 24 normal cervical epitheliums. GSE7803 included 10 normal squamous cervical epithelia samples, 7 high grade squamous intraepithelial lesions, and 21 invasive squamous cell carcinomas. Differential genes in the GSE9750 dataset were analyzed by GEO2R. Additionally, we analyzed the survival data from 197 cervical cancer patients in the TCGA database.

### Cell culture

The human cervical cancer cell lines HeLa, Caski and Siha were purchased from the American Type Culture Collection (ATCC, USA). All cells were cultured in RPMI-1640 medium (Gibco; Thermo Fisher Scientific, Inc) containing 10% FBS (Gibco; Thermo Fisher Scientific, Inc) and 1% penicillin-streptomycin (Gibco). These cells lived in an incubator maintained at 37 °C with 5% CO_2_.

### APMAP knockdown by shRNA lentivirus preparation

In terms of the APMAP knockdown, RNA interference target sequences were designed according to APMAP gene templates for constructing RNA interference lentivirus vectors. The GV115 lentivirus vector was used and the component sequence of lentiviral vectors was hU6-APMAP-CMV-EGFP. The RNA interference target sequence of APMAP was GAGTGACCTTCTTGATGCT, and the sequence of the control group was TTCTCCGAACGTGTCACGT. Lentiviral infections were performed at a multiplicity of infection of 5. To achieve the best experimental results, 48 h post-transfection was determined to be the best time point for infection according to the preliminary experiments when transfection efficiency, (based on the proportion of GFP fluorescing cells), was 70-80%. The successfully infected cells were utilized for subsequent experiments.

### Quantitative reverse transcription-polymerase chain reaction (qRT-PCR)

Trizol Reagent (Servicebio Biotechnology, China) is a common RNA extractant used to extract total RNA from all cell samples. Reverse transcription was performed using the ServiceBio® RT First Strand cDNA Synthesis Kit (Servicebio Biotechnology, China) and the real-time quantitative PCR reaction was then performed using specific primers on the SYBR Green real-time PCR Kit (Takara Biotechnology, China) and the ABI 7500 Real-Time PCR system (Applied Biosystems, Grand Island, NY, USA). The relative mRNA levels of the target genes were analyzed by 2^-ΔΔct^, and GAPDH was applied as an internal control. The primer sequences are shown in Table 1.

### Western Blotting

The total protein was extracted with RIPA lysate containing 1% phosphatase inhibitor and 1% protease inhibitor. Then, the BCA kit was utilized for quantitative protein analysis and the proteins were separated by SDS-PAGE at 8%, 10%, and 12%, according to the target molecular weight. The separated proteins were transferred to polyvinylidene fluoride (PVDF) membranes. The membranes were then floated in 5% skimmed milk with TBST for 1 h at room temperature. After sealing, the membranes were incubated in TBST containing specific antibodies, such as APMAP (1: 1000, Absin, China), GAPDH, β-catenin, E-cadherin, N-cadherin, vimentin, p-GSK3β, GSK3β, snail (1: 1000, cell Signal Technology, United States) at 4 °C overnight. Before and after the secondary antibody goat anti-rabbit antibody (1: 10000, cell Signal Technology, United States) was incubated at room temperature for 1h, the membranes were washed 4 times /5 min by TBST. The protein bands were indicated by the ServiceBio^®^ ECL chemiluminescence kit (Servicebio Biotechnology, China).

### Cell migration assay

The migration ability of HeLa, Caski and Siha cells was detected by Transwell assay and wound healing assay. For the Transwell assay, APMAP knockdown cells and negative control cells were added to the Transwell chamber of an 8 μm aperture filter membrane and diluted in 1640 medium without fetal bovine serum. Chambers containing 20,000 cells/200 μl were placed in 24-well plates with 500 ul 10% FBS 1640 medium per well. Each 24-well plate was then cultured in an incubator with 5% CO2 at 37 °C for 12h. After the chamber was taken out of the incubator, it was cleaned with PBS, fixed with formaldehyde, stained with 0.5% crystal violet, and photographed under an optical microscope. For the wound healing assay, APMAP knockdown cells and negative control cells were planted in a 12-well plate and a perpendicular 200-μl tips was used to create a wound when the cell fusion rate reached 90% or more. After removing the fragments, the cells were added to a serum-free medium to obtain some photos. The photographs were taken at fixed time points after 48h or 72 h of cell culture. All image data were analyzed by Image J.

### Cell apoptosis assay

Cell apoptosis was detected by Annexin V/Propidium Iodide (PI) double staining. 1×10^6^ collected APMAP knockdown cells and negative control cells were resuspended in 500 ul binding buffer and stained by 5 μl Annexin V-FITC and 5 μl PI for 30 min. Then, the cell apoptosis was observed with flow cytometry (BD Accuri™ C6; BD Biosciences).

### Statistical Analysis

All data were analyzed by SPSS 13.0 and GraphPad Prism 7.0 software and all experiments were repeated at least three times. Data analysis was indicated as mean ± standard deviation (SD). The comparison between the two groups was analyzed by the Student's t-test, and the comparison between multiple groups was analyzed by one way ANOVA. P <0.05 indicates a significant difference, P < 0.01 indicates a great significant difference.

## Results

### APMAP is highly expressed in cervical cancer tissues, and patients with high expression have poor survival prognosis

**Figure 1:**
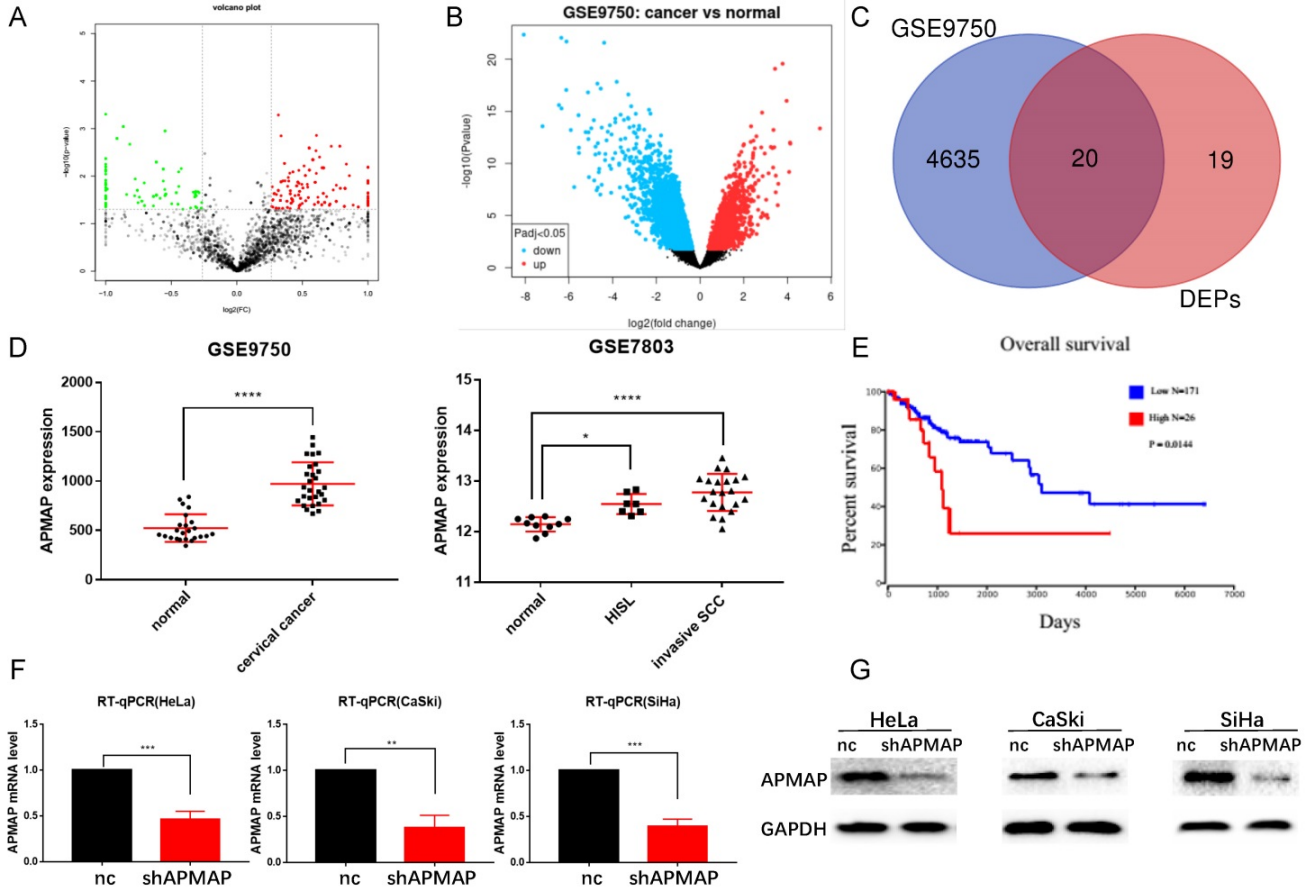
** The expression level and prognosis of APMAP. (A)** Volcanic maps of differential proteins. **(B)** Volcanic map of differential genes in the GSE9750 dataset. **(C)** Venn diagram of intersections of differential genes in A and B. **(D)** The mRNA expression of APMAP in the GSE9750 and GSE7803 datasets. **(E)** The prognosis of APMAP expression in TCGA cervical cancer patients. **(F-G)** RT-PCR and Western blotting showed that APMAP was knocked down by shRNA.

Proteomic analysis was conducted on both the cervical cancer tissues and paracancerous tissues of the three patients. Bioinformatics analysis of the original data revealed that there were 39 protein-coding genes with significant differences in expression between the cervical cancer and the paracancerous tissues (P < 0.01) (Figure 1A). 4655 differential genes were screened from the RNA sequencing data of the GSE9750 dataset (P < 0.01) (Figure 1B). There were 20 protein-coding genes in total (Figure 1C) where the two datasets intersected. From among these 20 genes, we decided to focus on APMAP, analyzing the expression of APMAP in normal tissues as well as in cervical cancer tissues in the GSE9750 and the GSE7803 datasets. The APMAP mRNA level was higher in cancer tissues than in normal cervical tissues (P < 0.05) (Figure 1D). For survival analysis, among the 197 cervical cancer patients in the TCGA database, patients with higher APMAP mRNA levels had a relatively shorter survival time compared to those with lower APMAP mRNA levels (P < 0.05) (Figure 1E). Then, we designed shRNA sequences for APMAP knockdown and applied RT-PCR and Western blotting to verify the knockdown efficiency. As shown in Figure 1F and 1G, the RNA and protein levels of APMAP showed a downward trend in the knockdown group.

### APMAP knockdown inhibits the migration of cervical cancer cells

**Figure 2:**
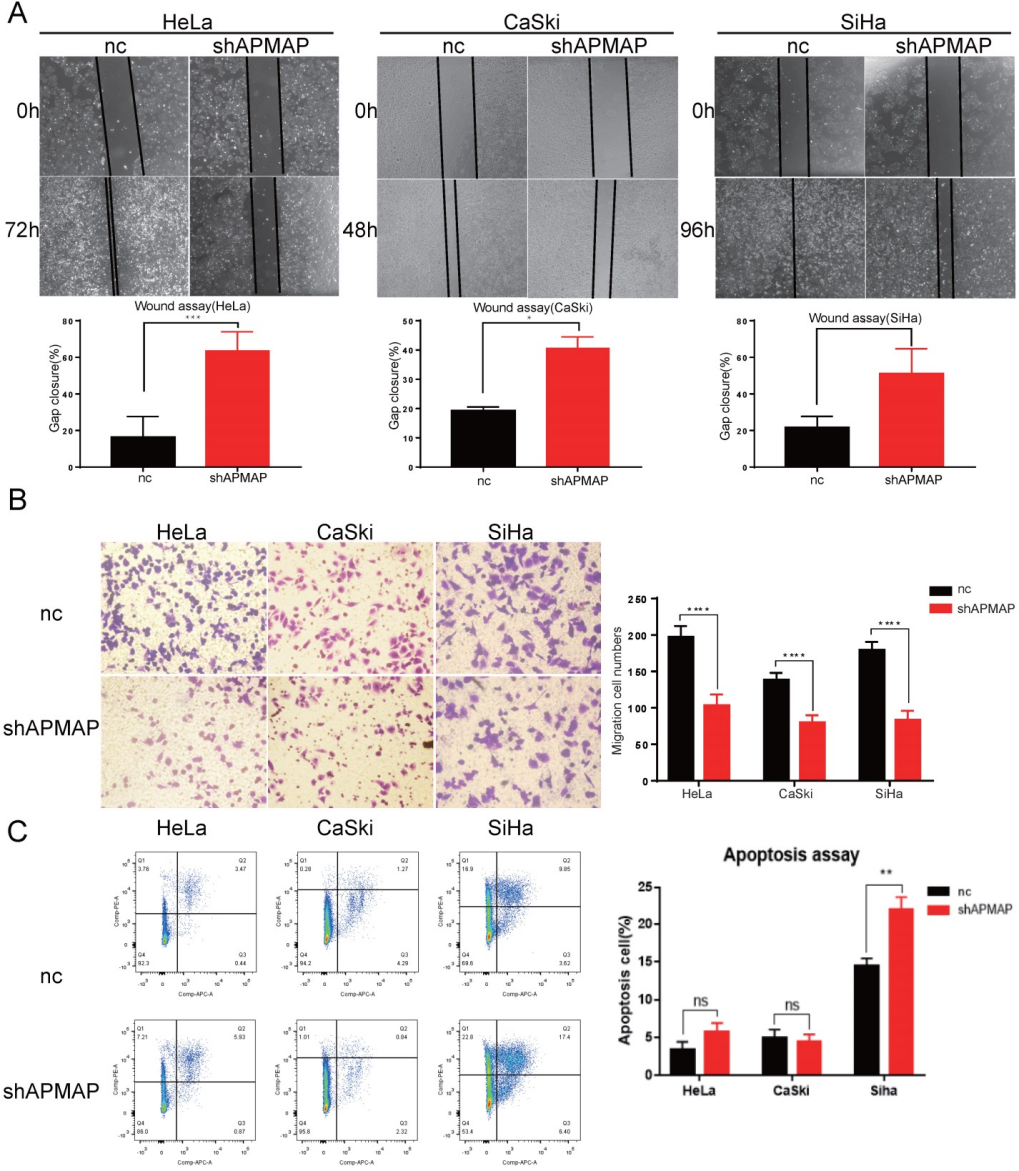
** APMAP promotes the migration of cervical cancer cells. (A,B)** The cell migration ability was detected by wound healing assay and Transwell assay. **(C)** The cell apoptosis ability was analyzed with flow cytometry.

To further assess the loss-of-function of APMAP, Transwell assay and the wound-healing assay were used to analyze cell migration. There was a significant difference in the migration ability between the APMAP knockdown group and the negative control group in HeLa, Caski and Siha cervical cancer cells. Gap cluster analysis of the wound healing experiment demonstrated that the APMAP knockdown group had poorer migration ability (Figure 2A). The Transwell results showed that the number of APMAP knockdown cells through the chambers was lower (Figure 2B). Additionally, we explored the effect of APMAP on the apoptotic ability of cervical cancer cells, using Annexin V/PI double staining to detect the number of apoptotic cells. We found the number of apoptotic cells did not show a significant increase after the APMAP knockdown. Only the Siha cells showed some statistically significant difference (P < 0.01) (Figure 2C). These results indicate that APMAP promoted the migration of cervical cancer cells but had little effect on cell apoptosis.

### APMAP activates the Wnt /β-catenin pathway and enhances the epithelial mesenchymal transition of cervical cancer cells

**Figure 3:**
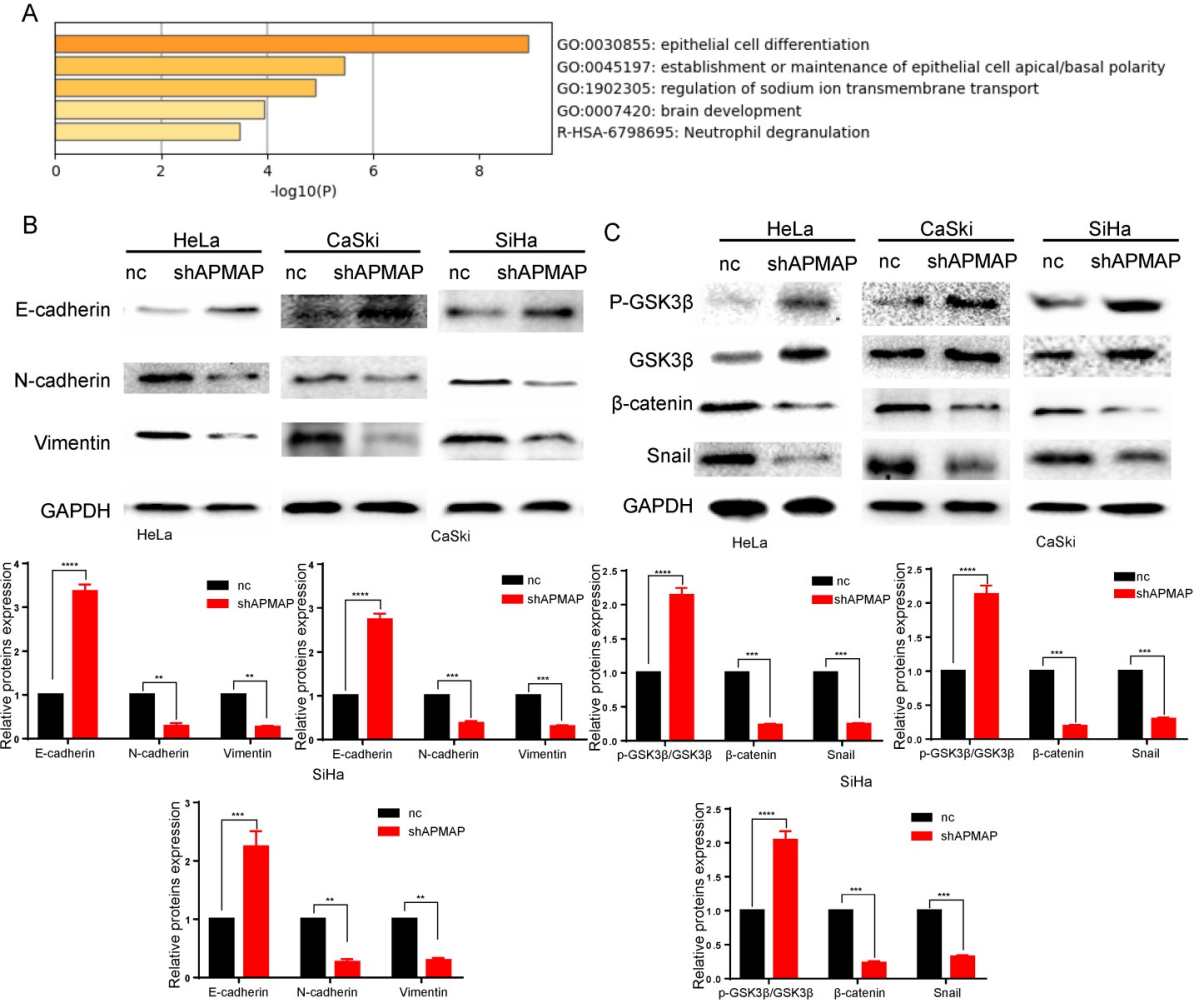
** Western blotting is applied to detect the epithelial-mesenchymal transition protein levels and the Wnt /β-catenin pathway markers in the APMAP knockdown group and the negative control group. (A)** Pathway and process enrichment analysis of 20 genes in Metascape database. **(B)** The protein expression of epithelial-mesenchymal transition markers. **(C)** The protein expression of Wnt/β-catenin pathway markers.

To analyze the main factors affecting the cervical cancer process, we performed pathway and process enrichment analysis on 20 differential genes in the Metascape database (http://metascape.org/) 23. The result demonstrates that epithelial cell differentiation played a major role (Figure 3A). The metastasis of cancer cells is closely related to epithelial mesenchymal transition. In the process of metastasis of cancer cells, epithelial cells lose cell adhesion characteristics, their cell polarity is disrupted, and they are subject to cytokeletal recombination. Drastic changes in morphology and reprogramming of gene expression also occur 24. Epithelial-mesenchymal transition related marker indexes mainly include such proteins as vimentin, N-cadherin, E-cadherin, and snail. By means of Western blot, we found the expression of these proteins was changed in APMAP knockdown group. The results showed that the protein levels of N-cadherin, vimentin and snail in the APMAP knockdown cells decreased, while the protein levels of E-cadherin increased (Figure 3B). APMAP knockdown also inhibited the Wnt/β-catenin pathway, one of the major pathways affecting epithelial-mesenchymal transition. Compared with the negative control group, the protein levels of β-catenin in the APMAP knockdown group decreased and the protein levels of p-GSK3β/GSK3β increased in the three types of cervical cancer cells (Figure 3C).

### The β-catenin agonists SKL2001 reverses the effect of APMAP knockdown in cervical cancer cells

**Figure 4:**
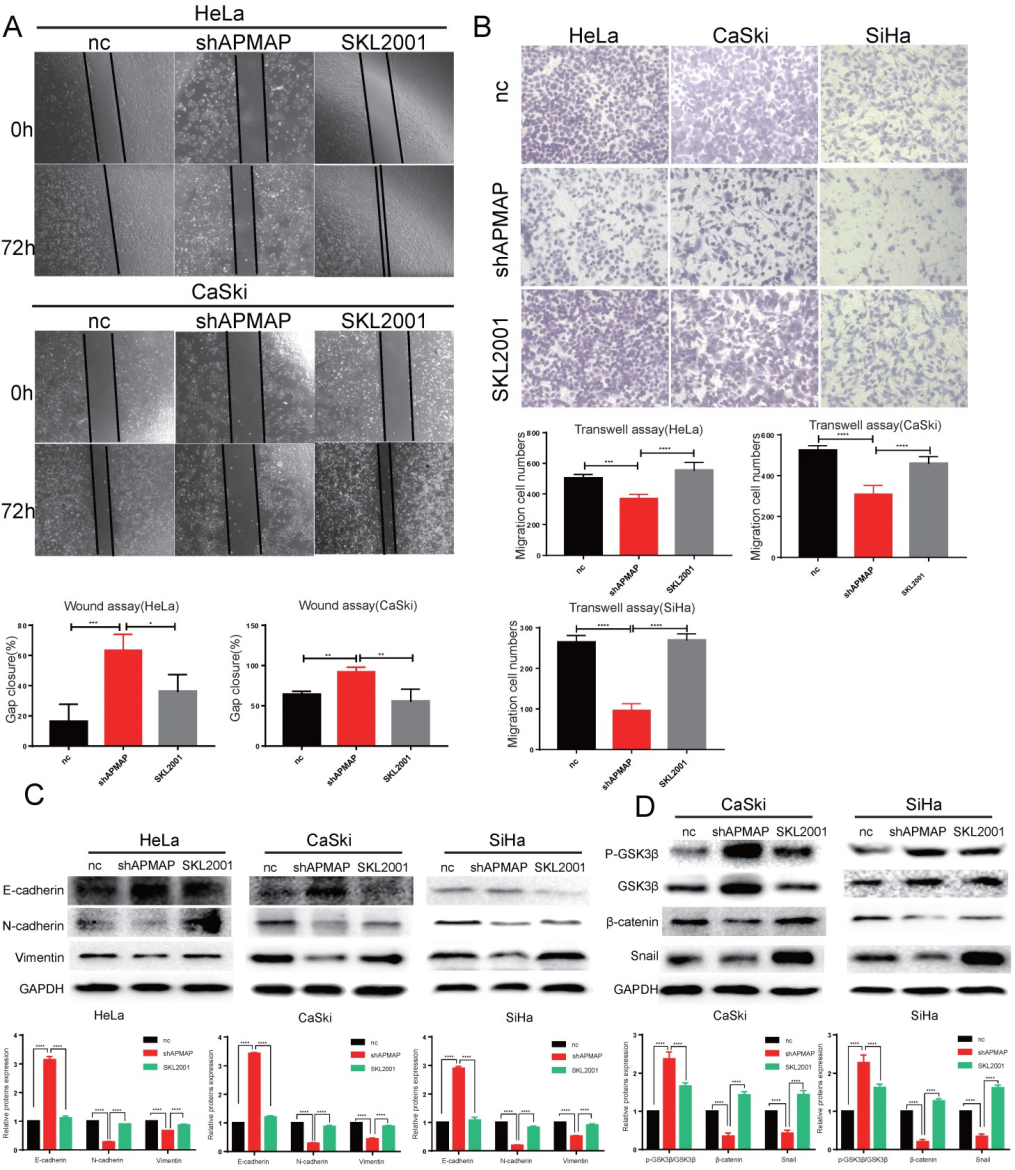
** SKL2001 recovers the inhibitory effect of APMAP knockdown. (A,B)** SKL2001 promotes the migration of APMAP knockdown cells. **(C,D)** SKL2001 activates the Wnt /β-catenin pathway and EMT of APMAP knockdown cells.

To verify whether APMAP promoted the migration and EMT of cervical cancer cells through the Wnt/β-catenin pathway, we used the β-catenin agonist SKL2001 to activate β-catenin in the APMAP knockdown cells (Figure 4D). After SKL2001 was added to the APMAP knockdown cells at 5μm, the cell migration ability and EMT were analyzed. Cell migrations were restored after the addition of SKL2001 (Figure 4A,B). Western blotting also detected N-cadherin, E-cadherin, vimentin and snail protein levels returned to normal (Figure 4C). And the β-catenin pathway was reactivated in the APMAP knockdown cells (Figure 4D). These rescue results indicated the Wnt/β-catenin pathway played a major role in APMAP promoting the migration of cervical cancer cells.

## Discussion

Cervical cancer remains one of the leading causes of death among women worldwide 25. Despite the clinical application of new therapeutic modalities, the prognosis of cervical cancer patients is still not ideal, with a 5-year survival rate of approximately 40% 26. One of the primary determinant affectings the prognosis of patients with cervical cancer is tumor metastasis. The invasion and metastasis of cervical cancer cells are also related to the interaction of multiple factors. Thus, it is of great significance to explore the invasion and metastasis of cervical cancer as well as its molecular mechanism so as to improve understanding of the molecular network of cervical cancer 27.

Growing evidence links obesity to various types of cancers 28. In this study, we identified that Adipocyte Plasma Membrane-Associated Protein (APMAP) was closely associated with cervical cancer cell migration. When APMAP was knocked down, the ability of the cervical cancer cells to migrate was greatly inhibited. In previous studies, APMAP was mainly associated with adipose differentiation 15, and there were few studies on its association with the occurrence and development of tumors 17, 19. However, APMAP is related to tumor metastasis. APMAP is upregulated by cholesterol and is involved in EMT progression as well as metastasis in prostate cancer. This suggests that APMAP is a key regulator providing the association between high-fat diet, obesity, and metastasis 19. Moreover, colorectal cancer cells with highly expressed APMAP were more likely to metastasize to the liver 17. In addition to the discovery that APMAP affects the migration of cervical cancer cells, we also found that APMAP was highly expressed in the cancer tissues of cervical cancer patients. Patients with high expression of APMAP had poor prognosis, while patients with low expression of APMAP had good prognosis, which indicates that APMAP can be used as a prognostic biomarker for patients with cervical cancer.

Epithelial mesenchymal transition plays a crucial role in the progression and metastasis of cervical cancer 29. We identified 20 differential genes including APMAP that were involved in epithelial cell differentiation during the progression of cervical cancer. Our Western blotting experiment revealed that APMAP knockdown inhibited the EMT process of cervical cancer cells, which decreased the protein expressions of snail, N-cadherin and vimentin, and increased the protein E-cadherin. It has been reported that snail could form positive feedback with β-catenin through physical interaction with β-catenin. The Wnt/β-catenin pathway is an important pathway regulating the EMT process 30, 31. For example, LGR5 acts as a glioma stem cell marker and promotes EMT through activating the Wnt/β-catenin pathway 32. We found that APMAP can activate the Wnt pathway. The addition of β-catenin agonist SKL2001 could reverse the effect of APMAP knockdown and restore the ability of cell migration and EMT process. These findings suggest that APMAP may influence the Wnt/β-catenin pathway in promoting the progression of cervical cancer.

In conclusion, for the first time, we explored the functional role and molecular mechanism of APMAP in cervical cancer. We identified that APMAP promotes cervical cancer cell migration and epithelial-mesenchymal transition by activating the Wnt/β-catenin pathway, which provides new understanding of the role of APMAP in tumor research.

## Figures and Tables

**Table 1:** The APMAP and GAPDH primer sequences

Gene	Primer
APMAP	Forward Primer CTGTCCTCCGAGACACCCAT
	Reverse Primer ACTTCCCTGGTCACAGTATCAT
GAPDH	Forward Primer TGACTTCAACAGCGACACCCA
	Reverse Primer CACCCTGTTGCTGTAGCCAAA

## Abbreviations

CC: cervical cancer
EMT: epithelial-mesenchymal transition
APMAP: Adipocyte Plasma Membrane Associated Protein
EPS15R: EGFR substrate 15 associated protein
MTT: 3-(4,5-dimethylthiazol-2-yl)-2,5-diphenyltetrazolium bromide
qRT-PCR: quantitative reverse transcription-polymerase chain reaction
SD: standard deviation
